# Reporting of Sociodemographic Variables in Randomized Clinical Trials, 2014-2020

**DOI:** 10.1001/jamanetworkopen.2021.10700

**Published:** 2021-06-02

**Authors:** Aaron M. Orkin, Gina Nicoll, Navindra Persaud, Andrew D. Pinto

**Affiliations:** 1 Department of Family and Community Medicine, University of Toronto, Toronto, Ontario, Canada; 2 Li Ka Shing Knowledge Institute, Unity Health Toronto, Toronto, Ontario, Canada; 3 Department of Psychology, University of Toronto, Toronto, Ontario, Canada; 4 Upstream Lab, MAP Centre for Urban Health Solutions, Li Ka Shing Knowledge Institute, Unity Health Toronto, Ontario, Canada; 5 Department of Family and Community Medicine, St Michael’s Hospital, Toronto, Ontario, Canada

## Abstract

This cross-sectional study assesses the frequency and thoroughness of reporting of sociodemographic variables in randomized clinical trials published in 5 high-impact health journals from January 2014 to July 2020.

## Introduction

Disease burden, health outcomes, and the effectiveness of interventions are associated with sociodemographic variables, such as race and ethnicity, income, and educational level. Understanding these social determinants of health and evaluating the effectiveness of health interventions among different sociodemographic groups is an essential approach to addressing health inequities.^1^

Randomized clinical trials (RCTs) provide the best available method to examine the causal effects of interventions in health care research. Such trials can serve the needs of racialized, low-income, and other marginalized and underserved communities only if sociodemographic variables and social determinants of health are measured and reported. It is unclear how frequently and how thoroughly the sociodemographic characteristics of participants are reported in RCTs. The objective of this study was to identify the frequency of reporting of sociodemographic variables in RCTs published in 5 high-impact health journals.

## Methods

A cross-sectional study was conducted of 10% of all RCTs published in 5 journals—*New England Journal of Medicine*, *JAMA*, *The BMJ, The Lancet*, and *Annals of Internal Medicine*—from January 1, 2014, through July 31, 2020. Every 10th article was retained from the search, starting with the most recently published.^2^ Each article was assessed by one of 4 reviewers (including G.N.) to identify the study’s medical discipline, intervention categories, study objective, and funding source and the sociodemographic variables used to characterize the population. Data queries were resolved by consensus. This study fell outside the scope of ethics committee review according to Canada’s Tri-Council Policy Statement on the ethical conduct for research involving humans. We followed applicable elements of the Strengthening the Reporting of Observational Studies in Epidemiology (STROBE) reporting guideline. The findings were tabulated to describe the sociodemographic variables reported in the included RCTs. All data analysis was conducted with Google Sheets spreadsheet software (Google).

## Results

During the study time frame, 2351 RCTs were published in the selected journals; 237 articles were randomly selected for review (Table 1). The most commonly reported sociodemographic variables were age (234 of 237 articles [98.7%]), any descriptor of sex or gender (234 of 237 [98.7%]), and race/ethnicity (115 of 237 [48.5%]) (Table 2). All other sociodemographic variables were reported in less than 15% of trials. Educational level or literacy was the next most commonly reported variable (34 articles [14.3%]). Income or socioeconomic status were rarely reported (14 [5.9%]). Three trials (1.3%) concerned interventions targeting social determinants of health, and 11 studies (4.6%) included participants based on a social determinant of health. Twenty-five studies (10.5%) were conducted in an all-male or all-female population; all of these trials studied diseases that affect only 1 sex (eg, prostate cancer, induction of labor). Six articles (2.5%) reported gender. No studies reported nonbinary gender descriptors.

**Table 1.** Random Sample of 237 Randomized Clinical Trials Published in *New England Journal of Medicine, JAMA, The BMJ, The Lancet,* and *Annals of Internal Medicine*, January 2014 to July 2020

Characteristic	Article, No. (%)
Journal	
* New England Journal of Medicine*	91 (38.4)
* The Lancet*	68 (28.7)
* JAMA*	50 (21.1)
* Annals of Internal Medicine*	16 (6.8)
* The BMJ*	12 (5.1)
Year of publication	
2014	35 (14.8)
2015	38 (16.0)
2016	36 (15.2)
2017	36 (15.2)
2018	36 (15.2)
2019	39 (16.5)
January-July 2020	17 (7.2)
Medical discipline	
Oncology	44 (18.6)
Cardiology	35 (14.8)
Infectious disease	29 (12.2)
Obstetrics and gynecology	16 (6.8)
Surgery	14 (5.9)
Respiratory medicine	12 (5.1)
Neurology	11 (4.6)
Endocrinology	10 (4.2)
Psychiatry	9 (3.8)
Other	57 (24.1)
Intervention	
Drugs	143 (60.3)
Surgery or radiotherapy	32 (13.5)
Prevention or screening	22 (9.3)
Rehabilitation and psychosocial	18 (7.6)
Communication, organization, education	9 (3.8)
Other	13 (5.5)
Funding source	
Public or government	80 (33.8)
Private or industry	106 (44.7)
Public and private	51 (21.5)
Continent^a^	
Europe	128 (54.0)
North America	120 (50.6)
Asia	85 (35.9)
Australia	59 (24.9)
South America	29 (12.2)
Africa	24 (10.1)

^a^ Includes some studies in multiple, overlapping categories.

**Table 2.** Reporting of Population Characteristics in a Sample of 237 Randomized Clinical Trials Published in *New England Journal of Medicine, JAMA, The BMJ, The Lancet,* and *Annals of Internal Medicine*, January 2014 to July 2020

Characteristic^a^	Articles, No. (%)							
	Total (N = 237)	2014 (n = 35)	2015 (n = 38)	2016 (n = 36)	2017 (n = 36)	2018 (n = 36)	2019 (n = 39)	2020 (n = 17)
Age	234 (98.7)	35 (100)	37 (97.4)	36 (100)	35 (97.2)	36 (100)	39 (100)	16 (94.1)
Sex or gender^b^	234 (98.7)	35 (100)	38 (100)	34 (94.4)	36 (100)	36 (100)	39 (100)	16 (94.1)
“Sex”^c^	228 (97.4)	32 (91.4)	38 (100)	33 (97.1)	36 (100)	35 (97.2)	38 (97.4)	16 (100)
“Male” only^c^	228 (97.4)	17 (48.6)	16 (42.1)	10 (29.4)	15 (41.7)	11 (30.6)	8 (20.5)	6 (37.5)
“Gender”^c^^,^^d^	228 (97.4)	3 (8.6)	0	1 (2.9)	0	1 (2.8)	1 (2.6)	0
Any nonbinary or nonbiological gender category	0	0	0	0	0	0	0	0
Race or ethnicity^e^	115 (48.5)	19 (54.3)	19 (50)	16 (44.4)	15 (41.7)	17 (47.2)	24 (61.5)	5 (29.4)
“White”	16 (13.9)	4 (22.2)	4 (21.1)	1 (6.3)	2 (13.3)	2 (11.8)	3 (12.5)	0
“White” and “non-White,” “other,” or any second category	17 (14.8)	1 (5.6)	1 (5.3)	0	5 (33.3)	6 (35.3)	4 (16.7)	0
≥3 Categories	72 (62.6)	11 (61.1)	10 (52.6)	14 (87.5)	8 (53.3)	9 (52.9)	15 (62.5)	5 (100)
Any indigenous category	17 (14.8)	2 (11.1)	1 (5.3)	2 (12.5)	0	2 (11.8)	6 (25)	4 (80)
“Other” category provided	64 (55.7)	10 (55.6)	8 (42.1)	12 (75)	8 (53.3)	11 (64.7)	13 (54.2)	2 (40)
Language	7 (2.9)	0	2 (5.3)	0	2 (5.6)	2 (5.6)	1 (2.6)	0
Educational level or literacy	34 (14.3)	7 (20)	2 (5.3)	5 (13.9)	5 (13.9)	8 (22.2)	5 (12.8)	2 (11.8)
Income or socioeconomic status	14 (5.9)	4 (11.4)	2 (5.3)	2 (5.6)	2 (5.6)	3 (8.3)	0	1 (5.9)
Employment status	14 (5.9)	2 (5.7)	2 (5.3)	1 (2.8)	3 (8.3)	4 (11.1)	2 (5.1)	0
Housing status	8 (3.4)	3 (8.6)	1 (2.6)	0	1 (2.8)	2 (5.6)	1 (2.6)	0
Health insurance status	10 (4.2)	2 (5.7)	2 (5.3)	0	2 (5.6)	1 (2.8)	3 (7.7)	0

^a^ Terms in quotation marks indicate the actual terminology used in the report.

^b^ Includes 28 articles that described the population in the text as all men or all women.

^c^ Sex or gender subcategories are reported as a percentage of 234 randomized clinical trials that reported any descriptor of sex or gender (n=234).

^d^ Refers to use of the term *gender* or any nonbiological descriptor of gender identity.

^e^ Race or ethnicity subcategories are reported as a percentage of 115 randomized clinical trials that reported any descriptor of race or ethnicity.

## Discussion

The findings of this cross-sectional study show that sociodemographic characteristics of study populations are minimally reported in RCTs published in high-impact journals. Descriptors of participant educational level or literacy were reported in only 14.3% of trials, and descriptors ofhousing, language, access to health care, or employment status appeared in less than 5% of studies.

White, male, and wealthy individuals are more often recruited and retained in RCTs compared with the general population or the population affected by the condition under investigation.^3,4,5^ The failure to gather or report on sociodemographic and social determinants of health obscures inequities in trial enrollment and outcomes. This threatens the applicability of trials, raises ethical concerns, and inhibits hypothesis building, subgroup analyses, and evidence syntheses.

This study has limitations. The articles included in the study represent a 10% sample of high-impact English-language publications and may not be representative of all RCTs. In addition, trialists may gather sociodemographic data but not report them.

All trial stakeholders share the responsibility to ensure that RCTs serve the health needs of the communities they are intended to serve. Randomized clinical trials cannot contribute to correcting health inequities if sociodemographic and social determinants are not measured and not reported. The Consolidated Standards of Reporting Trials–Equity 2017 statement calls for reporting sociodemographic variables but is only applicable to trials concerning health equity.^6^ For all trials, regulatory standards could help ensure that sociodemographic data are gathered and reported, whereas reporting guidelines and editorial standards could ensure that sociodemographic data are reported transparently.
